# The Secreted Immunoglobulin Domain Proteins ZIG-5 and ZIG-8 Cooperate with L1CAM/SAX-7 to Maintain Nervous System Integrity

**DOI:** 10.1371/journal.pgen.1002819

**Published:** 2012-07-19

**Authors:** Claire Y. Bénard, Cassandra Blanchette, Janine Recio, Oliver Hobert

**Affiliations:** 1 Department of Neurobiology, University of Massachusetts Medical School, Worcester, Massachusetts, United States of America; 2 Department of Biochemistry and Molecular Biophysics, Howard Hughes Medical Institute, Columbia University Medical Center, New York, New York, United States of America; University of California San Diego, United States of America

## Abstract

During nervous system development, neuronal cell bodies and their axodendritic projections are precisely positioned through transiently expressed patterning cues. We show here that two neuronally expressed, secreted immunoglobulin (Ig) domain-containing proteins, ZIG-5 and ZIG-8, have no detectable role during embryonic nervous system development of the nematode *Caenorhabditis elegans* but are jointly required for neuronal soma and ventral cord axons to maintain their correct position throughout postembryonic life of the animal. The maintenance defects observed upon removal of *zig-5* and *zig-8* are similar to those observed upon complete loss of the SAX-7 protein, the *C. elegans* ortholog of the L1CAM family of adhesion proteins, which have been implicated in several neurological diseases. SAX-7 exists in two isoforms: a canonical, long isoform (SAX-7L) and a more adhesive shorter isoform lacking the first two Ig domains (SAX-7S). Unexpectedly, the normally essential function of ZIG-5 and ZIG-8 in maintaining neuronal soma and axon position is completely suppressed by genetic removal of the long SAX-7L isoform. Overexpression of the short isoform SAX-7S also abrogates the need for ZIG-5 and ZIG-8. Conversely, overexpression of the long isoform disrupts adhesion, irrespective of the presence of the ZIG proteins. These findings suggest an unexpected interdependency of distinct Ig domain proteins, with one isoform of SAX-7, SAX-7L, inhibiting the function of the most adhesive isoform, SAX-7S, and this inhibition being relieved by ZIG-5 and ZIG-8. Apart from extending our understanding of dedicated neuronal maintenance mechanisms, these findings provide novel insights into adhesive and anti-adhesive functions of IgCAM proteins.

## Author Summary

The structure of nervous systems is determined during embryonic development. After this developmental patterning phase, active maintenance mechanisms are required to uphold the structural integrity of the nervous system. This concept was revealed through the genetic elimination of factors in the nematode *Caenorhabditis elegans,* which left the initial establishment of the nervous system during embryogenesis unperturbed, but subsequently resulted in postembryonic defects in its structural integrity. The extent to which such maintenance mechanisms exist, the nature of the players involved, and the mechanisms through which they operate are subjects of active investigation. In this study, we reveal two novel, previously uncharacterized maintenance factors encoded by the *zig-5* and *zig-8* genes. Both genes are predicted to encode small secreted immunoglobulin domains. We show that the two proteins operate by counteracting the anti-adhesive effects of a specific isoform of the SAX-7 Ig domain protein, the *C. elegans* homolog of L1CAM, a human protein involved in various neurological diseases. This study therefore provides novel mechanistic insights into nervous system patterning and may help to better understand the function of an important human disease gene.

## Introduction

The structural organization of an adult nervous system depends on two genetically separable processes. First, during development - the wiring phase - the soma and axonal/dendritic extensions of neurons need to be accurately positioned. This process depends on the precisely orchestrated activity of a multitude of well-characterized and dynamically acting guidance and signaling systems [1], [2], [3]. Second, during postembryonic life, dedicated maintenance factors ensure that neuronal soma, axon and dendrites maintain their precise position in neuronal ganglia and fascicles [4]. These maintenance factors counteract the various forms of mechanical and physical stress exerted onto a nervous system [4].

**Figure 1:**
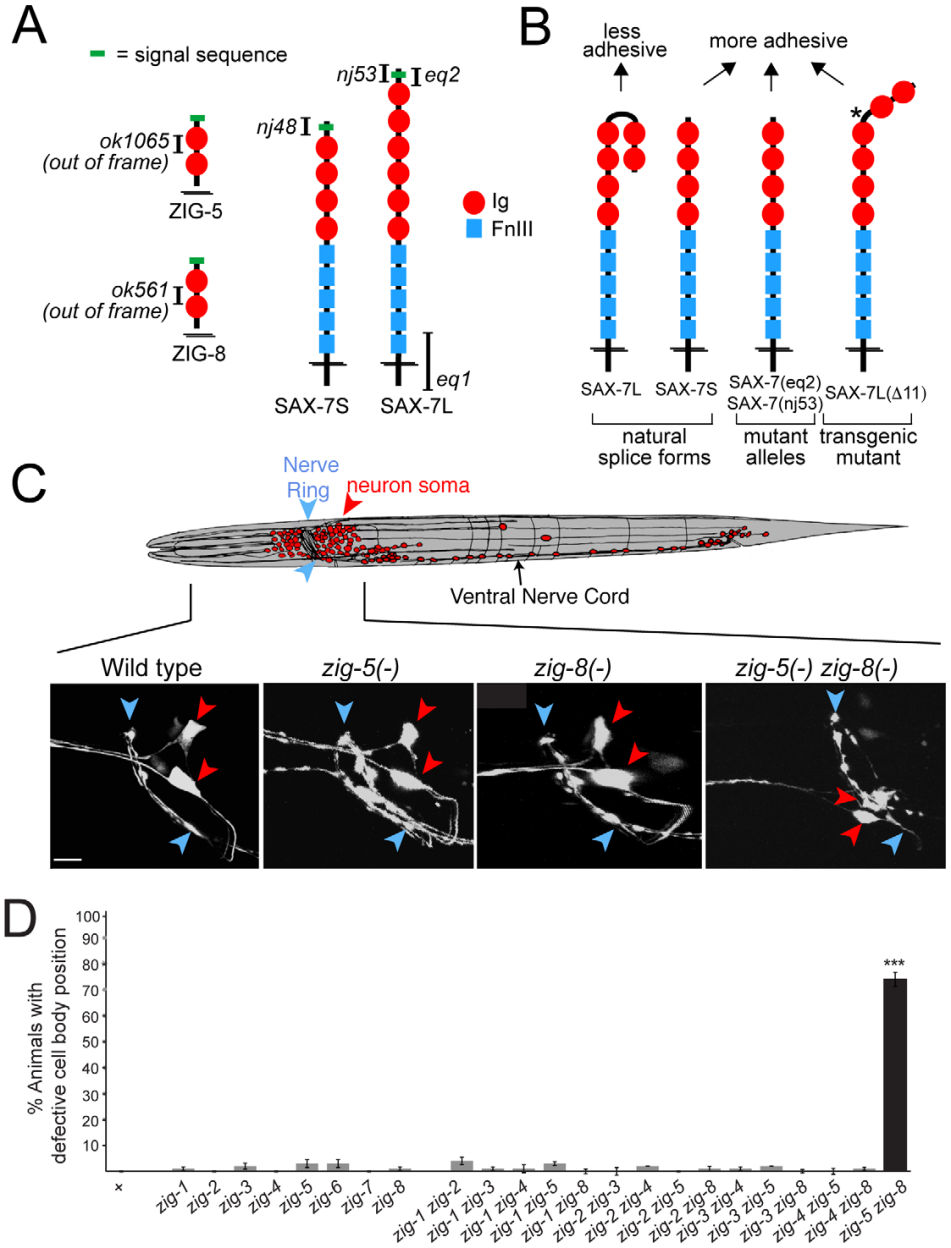
Neuronal maintenance factors and the defects caused by their removal. (A) Schematic protein structures and alleles used in this study. (B) Summary of previous *in vitro* and *in vivo* adhesion studies [6], [7]. Star indicates a shortened hinge region which prevents formation of the horseshoe configuration [7]. (C) ASI and ASH neuronal displacements observed in *zig-5(ok1065)* and *zig-8(ok561)* single and double mutant adult animals with the *oyIs14* reporter transgene. Blue arrowheads indicate position of the nerve ring and red arrowheads position of neuronal soma, which is scored relative to position of the nerve ring (wild type: behind nerve ring; mutant: on top of to nerve ring). Anterior to left, dorsal on top. Scale bar is 5 µm. (D) Quantification of ASI and ASH neuronal displacement in single and double mutants of the *zig* gene family. Alleles are described in [11]. Error bars indicate s.e.p.. Proportions of different animal populations were compared using the z-test. “*” indicates p<0.001.

The need for such maintenance mechanisms, and the specific maintenance factors involved, were first identified in the nematode *C. elegans*. The removal of a number of distinct molecules was found to result in no apparent effect on the initial positioning of neurons and fibers during embryonic development; yet the absence of these molecules affected the maintenance of the positioning of neuronal soma and fibers. These molecules include the L1CAM-type adhesion molecule SAX-7 [5], [6], [7], the extracellular matrix protein DIG-1 [8], a specific splice form the FGF receptor EGL-15, called EGL-15A [9] and ZIG-3 and ZIG-4, members of a family of small, secreted two-Ig domain proteins [10], [11](Figure 1A). While SAX-7, DIG-1, EGL-15A and the ZIG proteins appear to be solely dedicated to a maintenance role, other proteins, such as the basement membrane protein SPON-1/Spondin or UNC-70/β–Spectrin function both during the embryonic neuronal wiring phase and postembryonically in maintenance [12], [13].

How these maintenance factors functionally interact with one another has been unclear. In this paper, we describe the function of two previously uncharacterized ZIG proteins, ZIG-5 and ZIG-8, in maintaining neuron soma position. We tie their function specifically to the function of SAX-7, the *C. elegans* ortholog of the L1CAM family of vertebrate adhesion molecules. In *C. elegans*, SAX-7 exists in two splice forms, a short splice form (SAX-7S) and a long splice form (SAX-7L)(Figure 1B). Intriguingly, several studies have shown that the short isoform, SAX-7S, is more adhesive than the long isoform SAX-7L [6], [7], [14]. We show here that the two ZIG proteins ZIG-5 and ZIG-8 serve to prevent the SAX-7L isoform from interfering with cellular adhesion.

## Results

### *zig-5* and *zig-8* redundantly affect neuron soma and axon position

Loss of the *C. elegans* L1CAM ortholog *sax-7* affects the maintenance of neuron soma position in the main head ganglia of *C. elegans*, as well as the positioning of axons in the ventral nerve cord (VNC) [5], [6], [7], [14]. Loss of two members of the *zig* gene family (*zig-3* and *zig-4*) also affects the maintenance of axon positioning in the ventral nerve cord, but do not affect neuron soma position [10], [11]. To test whether *zig* genes may phenocopy the *sax-7* effect on the maintenance of soma position in head ganglia, we analyzed deletion alleles of all presently known, eight *zig* gene family members. Visualizing head neuron position either with *gfp* reporters or by dye labeling showed no defects in any *zig* single mutant strain (Figure 1C, 1D). Since *zig* genes may act redundantly, we generated double mutant combinations of all six neuronally expressed *zig* genes (that is all *zig* genes except muscle-expressed *zig-6* and *zig-7*; Aurelio and Hobert, 2002). This double mutant analysis led us to discover striking neuronal soma displacement defects in head ganglia of *zig-5(ok1065) zig-8(ok561)* double null mutant animals (Figure 1C, 1D). This defect can be observed both with cell-type specific *gfp* reporters (Figure 1C) as well as with dye filling of sensory neurons (47% animals affected; n = 150).

**Figure 2:**
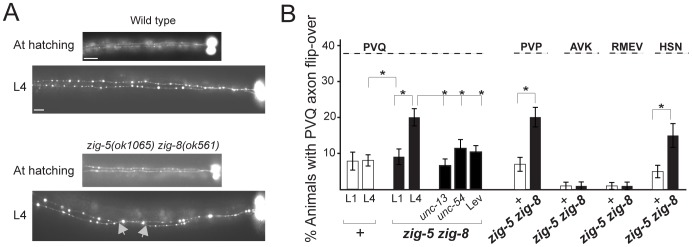
*zig-5* and *zig-8* affect axon positioning in the ventral nerve cord. (A) PVQ axon flip-over (red arrowhead) in *zig-5 zig-8* double mutants, observed first in later larval stages, but not earlier. Ventral view, anterior is to the left. Grey arrows indicate the location of axon flip-over. Scale bar is 5 µm. (B) Quantification of axon flip-over defects in *zig-5 zig-8* double mutants, using transgenes *oyIs14, hdIs29, bwIs2, oxIs12* and *zdIs13*, respectively. Single mutants *zig-5* and *zig-8* are wild type [11]. Animals were paralyzed with *unc* mutants or levamisole (Lev). Proportions of different animal populations were compared using the z-test. “*” indicates p<0.01.

*zig-5 zig-8* double mutants also display postembryonic axon position defects in the VNC (Figure 2). The *C. elegans* VNC is composed of unilaterally positioned motoneuron axons, located on the right side of the VNC and of axons of bilaterally symmetric neurons that extend along the left and right side of the VNC [15]. The left and right side of the VNC are separated by a midline structure, initially made of neuronal cell bodies, later of a hypodermal ridge [16]. In *zig-5 zig-8* double mutants, the axons of bilaterally symmetric neurons become aberrantly positioned across the midline during larval life (Figure 2A). Similar VNC axon defects can also be observed in *zig-3* and *zig-4* single mutant animals [10], [11]. Yet the cellular specificity of the VNC axon flip-over defects is broader in *zig-5 zig-8* double than in *zig-3* and *zig-4* single or double mutants, since HSN axons are affected only in *zig-5 zig-8* double mutants (Figure 2B). Other than these neuronal morphology defects, *zig-5 zig-8* double null mutant animals appear healthy, fertile, locomote normally and do not display other obvious morphological abnormalities. Also, the organization of muscle and epidermal tissues is normal in these mutants (assessed using specific reporters; data not shown), indicating that *zig-5* and *zig-8* function to maintain the integrity specifically of the nervous system.

The *zig-5 zig-8* double mutant phenotype can be rescued by genomic DNA clones that encompass these genes (Table 1) and can be phenocopied by RNAi (Table 2). Both *zig-5* and *zig-8* have previously been reported to be expressed in many neuronal and non-neuronal cell types in the head of the worm [*zig-5:* [10], *zig-8*: [17]], as well as the PVT tail neuron which extends its axons along the ventral nerve cord into the nerve ring [10]. The PVT neuron bears a critical role in conveying the function of *zig* gene family members in controlling maintenance of axon position in the ventral nerve cord [10], [11]. However, laser ablation of PVT does not affect head neuron position (0/57 PVT-ablated animals showed defects), and we therefore surmise that secretion of ZIG-5 and ZIG-8 from its many cellular sources in the head is required for maintaining cell body position. Even though we consider this model the most parsimonious based on the expression patterns of *zig-5* and *zig-8* in many head neurons, we have not been able to experimentally corroborate this notion as we observed no rescue of the mutant phenotypes by driving expression of *zig-5* and/or *zig-8* under control of variety of different heterologous promoters (tested promoters: neuronal *unc-14, osm-6, sra-6,* muscle *myo-3,* hypodermis *dpy-7*; injected at different concentrations from 0.1 to 125 ng/µL) and with different co-injection markers (pRF4, *ttx-3*::mCherry). Heterologous expression of *zig-5* and *zig-8* (under *unc-14, myo-3* and *dpy-7* promoter; 3 lines each) do not induce defects in a wild-type background, indicating that the lack of rescue through heterologous expression is not the result of overexpression. Since rescue with genomic clones is also just partial, it is conceivable that the correct dosage of *zig-5* and *zig-8* is critical for their function, yet difficult to achieve in transgenic animals. We note that we have also have problems expressing ZIG-5 and ZIG-8 in heterologous tissue culture cells.

**Table 1:** Rescue of *zig-5 zig-8* mutant phenotypes.

Genotype	Displaced ASH/ASI soma^1^	n
wildtype	0%	>400
*zig-5(ok1065)*	3%	150
*zig-8(ok561)*	1%	150
*zig-5(ok1065) zig-8(ok561)*	74%^2^	265
*zig-5(ok1065) zig-8(ok561); Ex[zig-5* fosmid]; *line #1*	45% (p<0.001)^3^	97
*zig-5(ok1065) zig-8(ok561); Ex[zig-5* fosmid]; *line #2*	39% (p<0.001)^3^	99
*zig-5(ok1065) zig-8(ok561); Ex[zig-8* YAC]; *line #3*	35% (p<0.001)^3^	176
*zig-5(ok1065) zig-8(ok561); Ex[zig-8* YAC]; *line #4*	42% (p<0.001)^3^	130

1 On top of or anterior to nerve ring. Scored with *oyIs14 (sra-6::gfp)*.

2 Repeated from Figure 1 for comparison.

3 Compared to non-transgenic control (74% defective). Injection of *zig-5* and *zig-8* expressed under the control of a number of neuronal or pharyngeal promoters, at a range of concentrations, did not produce better rescue of the mutant phenotypes than that obtained with the fosmid and YAC.

**Table 2:** *zig-5 zig-8* neuron position defects are maintenance defects.

Genotype	Timing of RNAi	Displaced ASH/ASI soma^1^	n
wildtype	none	0%	>400
*zig-5(ok1065) zig-8(ok561)*	none	74%^2^	265
Control RNAi	constitutive (P0 and F1)^3^	1%	84
	P0 only: F1 onwards	2%	102
	P0 only: L4 onwards	2%	63
	P0 only: Young adult onwards	0%	91
*zig-5(RNAi) zig-8(RNAi)*	constitutive (P0 and F1)^3^	9%^2^ (p<0.05)^4^	149
	P0 only: L1 onwards	6% (p<0.05)^4^	119
	P0 only: L4 onwards	7% (p<0.05)^4^	75
	P0 only: Young adult onwards	6% (p<0.05)^4^	63

All animals were scored as 3-day-old adults.

1 On top of or anterior to nerve ring. Scored with *oyIs14 (sra-6::gfp)*.

2 Repeated from Figure 1 for comparison.

3 Refers to P0 and F1 generation.

4 p values calculated compared to control RNAi ( = empty vector).

### *zig-5* and *zig-8* maintain neuroanatomy

**Figure 3:**
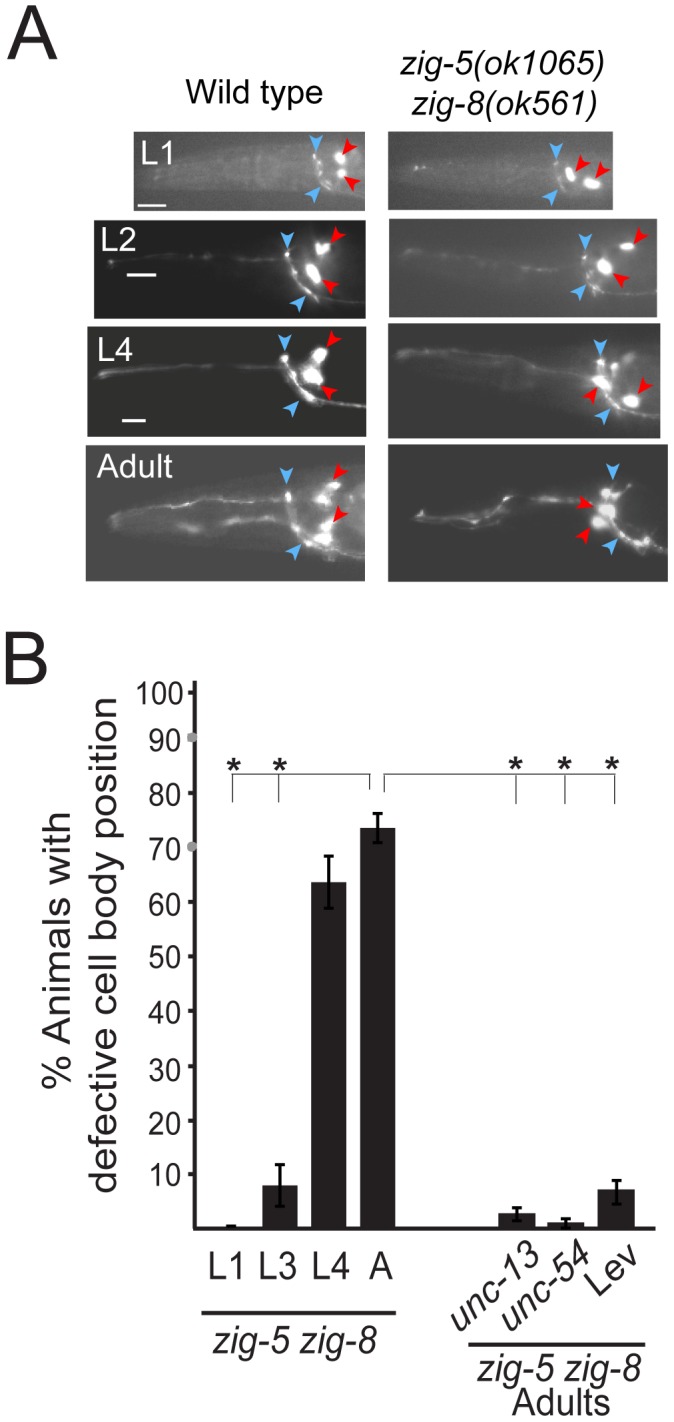
The *zig-5* and *zig-8* soma positioning defects are maintenance defects. (A) Soma displacement defects in *zig-5 zig-8* animals are only apparent at late larval and adult stages. See legend to Figure 1C for explanation of symbols. *Scale* bar is 5 µm. (B) Quantification of ASI and ASH neuronal displacement in *zig-5 zig-8* double mutants at different developmental stages and when paralyzed in *unc* mutant backgrounds or on the drug levamisole (Lev). Proportions of different animal populations were compared using the z-test. “*” indicates p<0.001.

As in the case of *sax-7* mutants [5], [6], [7], [14], the neuronal defects of *zig-5 zig-8* double null mutants appear to be reflective of maintenance rather than developmental defects. First, the neuronal soma position defects of *zig-5 zig-8* double mutants manifest themselves only postembryonically, long after birth and initial placement of neuronal cell bodies in the embryo (Figure 3). That is, animals in early larval stages appear completely indistinguishable from wild type and the phenotype manifests itself fully only in adult animals (Figure 3). Likewise, the axons of PVP and PVQ are initially positioned normally along the ventral midline during embryogenesis, but later become displaced during larval growth (Figure 2B). Second, the soma position defect can be evoked also in wild-type animals upon postembryonic knockdown of *zig-5* and *zig-8* by RNAi (Table 2). Third, the soma and axon displacement defects of the *zig-5 zig-8* double null defects can be suppressed through prevention of locomotion of the animal, achieved either by genetic means or through drug treatment (Figure 2B, Figure 3B). A similar suppression of maintenance defects is a hallmark of all previously described maintenance mutants [6], [8], [10], [11] and indicates that ZIG-5 and ZIG-8, like other maintenance factors, serve to counteract the effects of physical movement, enabling neuronal soma and axonal projection to appropriately maintain their position.

### Loss of *zig-5* and *zig-8* results in aberrant SAX-7L activity

**Figure 4:**
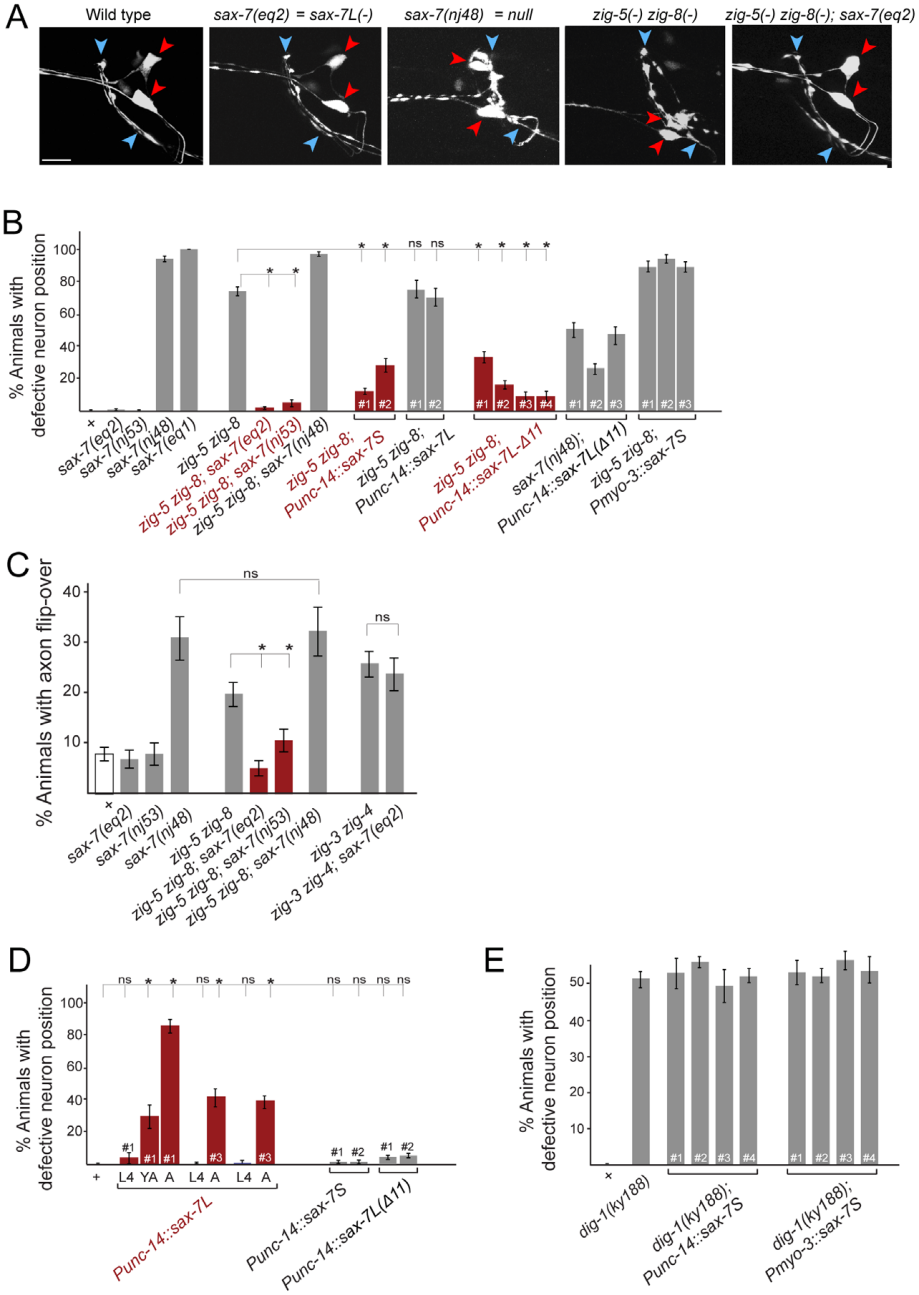
Genetic interactions between *zig-5, zig-8* and *sax-7*. (A) ASI and ASH neuronal displacements in mutant adult animals scored with the *oyIs14* reporter transgene. Wild-type and *zig-5 zig-8* double mutant pictures are the same as shown in Figure 1C and shown for comparison only. See legend to Figure *1C* for explanation of symbols. *Scale* bar is 5 µm. (B) Quantification of ASI and ASH neuronal position in genetic different backgrounds. “punc-14” is a panneuronal driver. *Punc-14::sax-7L*: DNA was injected at 75 ng/uL (line 1) or 50 ng/uL (lines 2 and 3). *Punc-14::sax-7S and Punc-14::sax7Δ11*: DNA was injected at 50 ng/uL. Proportions of different animal populations were compared using the z-test. “*” indicates p<0.001. (C) Genetic interactions of *zig-5, zig-8* and *sax-7* in controlling axon positioning in the ventral nerve cord. Quantification of PVQ axon flip-overs with transgene *oyIs14*. Error bars indicate s.e.p. Proportions of different animal populations were compared using the z-test. “*” indicates p<0.01. (D) Effect of ectopic expression of various forms of *sax-7* in a wild-type background. ASI and ASH neuronal position are quantified. Proportions of different animal populations were compared using the z-test. “*” indicates p<0.001. (E) Quantification of ASI and ASH neuronal position in *dig-1(ky188)* mutant animals. “*Punc-14*” is a panneuronal driver for expression of SAX-7S. There are no statistically significant differences between *dig-1* animals and any of the transgenic *dig-1* animals expressing *sax-7S.*

The *zig-5 zig-8* double null mutant phenotype, both in terms of the head soma positioning and ventral cord axon positioning defects, is similar to the null phenotype of *sax-7* (Figure 4A, 4B). We set out to test whether these loci act in the same pathway by examining whether combinations of null alleles show similar phenotypes (which would argue for acting in the same pathway) or enhance each other's phenotype (which would argue for acting in separate pathways). While the null phenotype of *sax-7* is completely penetrant for the soma positioning defect, the axon positioning defect is only partially penetrant, therefore allowing to do the genetic interaction test of *sax-7* and the *zig* genes. We find that the *sax-7(nj48)* null mutant phenotype is not enhanced in *zig-5 zig-8; sax-7* triple mutant animals, suggesting that these genes act in a similar genetic pathway (Figure 4C).

To further investigate the genetic interaction of *zig-5, zig-8* and *sax-*7, we considered different isoforms of the *sax-7* locus. *sax-7* produces two distinct splice forms, a longer isoform, *sax-7L,* that displays the canonical, L1CAM-type 6 Ig/5 FnIII-domain architecture and a shorter isoform, *sax-7S*, which lacks the first two Ig domains [6], [14] (Figure 1A). Cell aggregation assays in tissue culture as well as transgenic rescue experiments with the two different isoforms have firmly established that the short isoform, *sax-7S*, is more adhesive than the long isoform, *sax-7L* [6], [7], [14]. Based on structural studies of various IgCAM proteins, including L1CAM family members themselves, the first 4 Ig domains of SAX-7L are expected to be configured in a horseshoe conformation in which Ig1 and Ig2 fold back onto Ig3 and Ig4 (Figure 1B) and it is this horseshoe configuration that is thought to engage in homophilic interactions [18], [19], [20], [21], [22], [23], [24]. It is therefore curious that the SAX-7S form, as well as a mutant version of SAX-7L that is unable to adopt the horseshoe configuration (through shortening of the hinge region between Ig2 and Ig3, “SAX-7L(Δ11)”; Figure 1B), is more adhesive than the presumably horseshoe-configured, wild-type SAX-7L protein [6], [7], [14]. Further, consistent with the first two Ig domains being dispensable for effective homophilic adhesion, two alleles, *eq2* and *nj53,* that exclusively disrupt the *sax-7L* form, but not the *sax-7S* form [6], [14], neither affect soma nor axon position (Figure 4B; see Figure 1A for schematic presentation of the *sax-7* isoform specific alleles).

Unexpectedly, we find that the two *sax-7L*-specific alleles each completely suppress the soma displacement defect of *zig-5 zig-8* double mutants: While three quarters of *zig-5 zig-8* double mutants display soma positioning defects, almost no triple mutant animals do (Figure 4A, 4B). Similarly, the *sax-7L* isoform specific alleles also suppress the VNC axon defects of *zig-5 zig-8* double mutants (Figure 4C). These results indicate that *zig-5* and *zig-8* function is not required (i.e. their loss produces no phenotype) as long as the *sax-7L* isoform is not present. In other words, in wild-type animals, SAX-7L has the potential to disrupt cell adhesion, but this disruptive ability is counteracted by *zig-5* and *zig-8*. This disruptive function is uncovered in *zig-5 zig-8* mutants.

To further probe the disruptive activity of SAX-7L, we tested whether expression of SAX-7L above its usual level in an otherwise wild-type background may cause soma and axon positioning defects. Using a pan-neuronal *unc-14* driver to express *sax-7L*, we indeed find such transgenic animals display severe soma and axon position defects that are virtually indistinguishable from a complete loss of *sax-7L/S* function or loss of *zig-5* and *zig-8* function (Figure 4D shows the soma defect; the axon defect is 58% penetrant, n = 86). The *sax-7L* overexpression-induced defects are maintenance defects as they are only evident in late larval and adult stages, but not earlier, after hatching (Figure 4D). Overexpression of *sax-7S* using the same driver does not induce any defects and neither does the *sax-7L* isoform when it is converted into a more adhesive form through shortening of the hinge region between Ig2 and Ig3 (*sax-7L(Δ11)*, as shown in Figure 1B) (Figure 4D). This result can be interpreted to mean that overexpression of SAX-7L overwhelms the ability of ZIG-5/ZIG-8 to convert SAX-7L to a more adhesive form, thereby revealing the disruptive function of SAX-7L. However, the overexpression effect of SAX-7L is not alleviated in animals that carry arrays with extra copies of *zig-5* and *zig-8*, but this experiment is not easily interpretable in light of the issues that we discuss above with transgenic *zig-5* and *zig-8* expression.

We considered the possibility that the disruptive function of SAX-7L, which is revealed upon removal of *zig-5* and *zig-8*, lies in opposing the adhesive function of SAX-7S. To test this possibility, we examined whether overexpression of SAX-7S may overcome the antagonistic function of endogenous SAX-7L and therefore may abrogate the need for *zig-5* and *zig-8*. To this end, we generated transgenic animals that overexpress *sax-7S* in a *zig-5 zig-8* double mutant background. We find that pan-neuronal *sax-7S* expression completely rescues the soma positioning defects of *zig-5 zig-8* double mutants (Figure 4B). Expressing *sax-7S* from a muscle specific promoter does not rescue the *zig-5 zig-8* defects (Figure 4B). Neuronal overexpression of the long isoform *sax-7L* also does not rescue the *zig-5 zig-8* defects (Figure 4B). However, if the *sax-7L* isoform is converted into a more adhesive form through shortening of the hinge region between Ig2 and Ig3 (*sax-7L(Δ11)*, as shown in Figure 1B), *sax-7L* becomes able to rescue the *zig-5 zig-8* double null mutant phenotype (Figure 4A, 4B). We therefore conclude that providing additional copies of non-horseshoe-configured *sax-7* can compensate for the loss of *zig-5* and *zig-8*. This compensatory effect is not simply caused by the addition of unspecific “glue”, since SAX-7S is not able to rescue the neuronal soma position defects of animals lacking the *dig-1* maintenance factor (Figure 4E).

**Figure 5:**
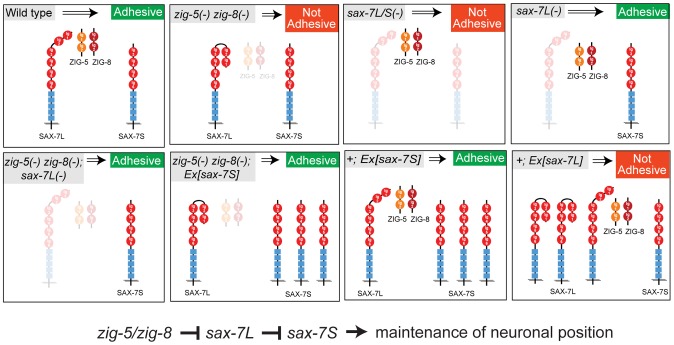
Genetic interactions of *zig-5, zig-8* and *sax-7*. This figure summarizes the genetic interaction data described in this paper.

In conclusion, our results suggest that ZIG-5 and ZIG-8 exert their activity on neuronal architecture through their genetic interactions with SAX-7. All genetic interaction tests are schematically summarized in Figure 5.

### The interaction of ZIG proteins with SAX-7 is context-dependent

The head neuron position defects described above concern neurons in the most populated ganglia of the head, the two lateral ganglia which containing about 50 neurons. In the smaller ventral head ganglion, the adhesive function of SAX-7 may be regulated in a different manner. This is because the soma positioning defects of the two ventral ganglion neuron types AIY and AVK, that are observed in *sax-7* null mutant animals [7], are not phenocopied in *zig-5 zig-8* double null mutant animals (0/50 animals show defects). Yet, as in neurons of the lateral ganglia, the *sax-7* defect again is more efficiently rescued by *sax-7S* compared to *sax-7L* [7]. Conceivably, another combination of *zig* genes may act to promote *sax-7* gene function in this cellular context.

We also examined the reverse and asked whether *zig* gene function can generally be explained *zig* genes affecting *sax-7* gene function. To this end we turned to *zig-3 zig-4* double mutants in which head soma position is unaffected, but positioning of axons in the ventral nerve cord fails to be maintained [11]. Similar axon maintenance defects are observed in animals lacking *sax-7* [7], but are not observed in the *sax-7L-*specific allele *eq2* (Figure 4C). In this case, *eq2* is not able to suppress the *zig-3 zig-4* double mutant phenotype (Figure 4C). However, as mentioned above, *eq2* can suppress the axon positioning defects of *zig-5 zig-8* double mutants (Figure 4C). In conclusion, the interaction of *zig* genes and *sax-7* depends on the type of ZIG proteins that evoke the defects and it depends on cellular context, possibly because the adhesive substrate for specific neuronal ensembles may differ at distinct locations in the worm.

## Discussion

Our analysis has revealed the function of two previously unstudied, secreted ZIG proteins, ZIG-5 and ZIG-8, thereby further broadening the concept of the requirement of specific factors for maintaining neuronal anatomy. Together with the previously characterized *zig-3* and *zig-4* genes, four of the eight presently known *zig* genes have now been specifically implicated in nervous system maintenance. The spectrum of activities of these four *zig* genes is partially overlapping (in the VNC), but also distinct (soma positioning in head ganglia). Given these precedents, it is conceivable that the remaining *zig* genes also have functions in maintaining nervous system architecture, perhaps affecting distinct subset of ganglia or even just individual neurons which have so far escaped attention.

Moreover, we have provided first hints toward the mechanism, but also diversity of ZIG protein function, by demonstrating that two ZIG family members (*zig-5* and *zig-8*, but not *zig-3* or *zig-4*) genetically interact with an IgCAM protein, the L1CAM protein SAX-7. These interaction results are summarized in Figure 5. Given the involvement of L1CAMs in various neurological diseases, a detailed understanding of this family of proteins is much warranted [25]. We have provided here novel and unexpected insights into mode of regulation of the adhesive activities of the L1CAM protein SAX-7.

We found that the likely horseshoe-configured SAX-7L isoform of SAX-7, previously shown to provide less homophilic adhesiveness than the shorter isoform SAX-7S [6], [7], has in fact an anti-adhesive activity in an *in vivo* context. This anti-adhesive activity is counteracted in wild-type animals by the two ZIG proteins ZIG-5 and ZIG-8, and thereby revealed only by either removal of ZIG-5 and ZIG-8 or by overexpression of SAX-7L. SAX-7L may engage SAX-7S in multimers in *cis* that are not able to engage in homophilic interactions in *trans*. ZIG-5 and ZIG-8 may be able to break up those complexes, for example by opening the SAX-7L horseshoe configuration, thereby converting SAX-7L into a more adhesive state and/or freeing up SAX-7S, which can then engage in homophilic *trans* interactions. *In vitro* cell aggregation assays, which we have not been able to undertake so far due to our inability to heterologously produce ZIG proteins, may address these possibilities in the future.

The *in vivo* studies on SAX-7 protein function have yielded results that are unexpected if one considers that several *in vitro* studies have provided evidence for horseshoe-configured IgCAMs engaging in homophilic interactions [21], [22], [23], [24]. The SAX-7 case argues for additional and alternative types of homophilic interactions of IgCAM molecules that are not only independent of a horseshoe configuration but also more adhesive than the horseshoe configuration. SAX-7S may be able to engage in other versions of previously described IgCAM interactions, such as the zipper mechanism proposed for the IgCAM superfamily member NCAM [26]. It is also conceivable that the adhesive mechanisms of IgCAM proteins may have diverged in the course of evolution and that the SAX-7/ZIG adhesion pathway may not be phylogenetically conserved.

Interestingly, while the activity of *zig-5* and *zig-8* can be explained entirely through their effect on *sax-7* (as best evidenced by the complete suppression of the *zig-5 zig-8* double mutant phenotype by *sax-7L-*specific alleles), an interaction with *sax-7* is not the only way the ZIG family members operate. First, the genetic interactions of *zig-5* and *zig-8* with *sax-7* are apparent in some ganglia and axonal tract, but not others. And second, the mutant phenotype of *zig-3* and *zig-4,* even though similar to those of *zig-5* and *zig-8* in the context of the VNC, shows no genetic interaction with the *sax-7L* isoform. The existence of other maintenance factors, such as the extracellular matrix proteins DIG-1, F-spondin or the FGF receptor EGL-15 all point to a diversity of mechanisms involved in maintenance of nervous system integrity [4].

On a mechanistic level, our findings support the hypothesis [4] that maintenance of tissue integrity is controlled through a finely tuned and tightly regulated balance of adhesive and anti-adhesive forces (Figure 5). The function of SAX-7L may lie in modulating the strongly adhesive function of SAX-7S at stages and in tissues where high adhesiveness is not desired. This anti-adhesiveness of SAX-7L may in turn be eliminated by ZIG proteins when high degree of adhesiveness is required, such as during postembryonic life to maintain neuron position. It will be interesting to see whether modulation of the homophilic interaction of IgCAM proteins through interaction with other Ig domain proteins is a common theme in maintenance of neuronal positioning. The wealth of Ig domain proteins, many of them secreted, in vertebrate and invertebrate genomes [27], suggests that many such modulatory interactions remain to be discovered.

## Materials and Methods

### Strains and transgenes

The nature and structure of all *zig* and all *sax-7* mutant alleles were previously described in detail [6], [11], [14] and some of them are schematically shown in Figure 1A. Genotyping was done by PCR. *sax-7* transgenes are described in [7]. Transgenes used to rescue *zig-5 zig-8* double mutant phenotype were obtained by injecting fosmid WRM063cG06 containing the *zig-5* gene (injected at 20 ng/µL along with *ttx-3::mCherry* at 100 ng/µL) and the YAC Y39E4B, containing the *zig-8* gene (injected at 10 ng/µL along with pRF4 *rol-6(su1006d)* at 100 ng/µL).

### Scoring of neuroanatomy

The following *gfp* transgenes were used to score anatomy, using a Zeiss Axioplan 2 microscope: *oyIs14*: *Is[sra-6::gfp]*, *hdIs29: Is[sra-6::DsRed2; odr-2::gfp] oxIs12 Is[unc-47::gfp], bwIs2 Is[flp-1::gfp]* (described in [11]. Cell body position was examined in three- to five-days old adults, i.e. worms that have lived for 3 to 5 days after the L4 to young adult molt. The position of the soma of ASI and ASH neurons is normally posterior to the nerve ring, and was scored defective when the cell body of at least one neuron was anterior to the nerve ring, on top of it, or contacting it.

Ventral nerve cord anatomy was examined in freshly hatched L1 larvae (<30 min post-hatch) and in L4 larvae. The axons of the pairs of bilateral neurons examined are normally separate and lie on either side of the ventral midline. An axon was scored defective when a segment of its length was located on the opposite side of the ventral nerve cord and contacted the axon on the other side.

For the paralysis on levamisole experiment, embryos were placed on plates containing 50 µM levamisole and seeded with OP50 bacteria, were allowed to reach the L4 and adult stages, and were examined as above.

All phenotypes were scored as percent animals defective and results are shown with error bars representing the standard error of proportion. Statistical significance was calculated using the z-test to compare the proportion of abnormal animals of two genotypes. When using the same control for multiple comparisons, the P value was multiplied by the total number of comparisons.

### RNA interference

*oyIs14* L4 hermaphrodites were placed on bacteria harboring plasmids to express dsRNA corresponding to the genes *zig-5* and *zig-8* (J. Ahringer library), or the empty vector (L4440). The inserts for the *zig-5* and *zig-8* plasmids were verified by sequencing. The two bacterial strains containing the *zig-5* and the *zig-8* plasmids were grown separately overnight. Bacterial cultures were concentrated by centrifugation, mixed and added to RNAi plates. A day later, these animals were transferred onto fresh plates containing the RNAi bacteria. F1 animals were scored for the morphology of the chemosensory neurons at days 3–5 of adulthood. Animals were also placed on RNAi plates at the L1, L4 and young adult stages, and their neuroanatomy was examined when they reached 3–5 days of adulthood. Similar experiments were carried out in the *rrf-3;oyIs14, eri-1;lin-15b;oyIs14* genetic backgrounds, as well as with RNAi of *zig-5* in *zig-8* mutant background, and vice versa, but failed to elicit stronger defects.

## References

[1] Tessier-Lavigne M, Goodman CS (1996). The molecular biology of axon guidance.. Science.

[2] Kolodkin AL, Tessier-Lavigne M (2010). Mechanisms and Molecules of Neuronal Wiring: A Primer..

[3] Yu TW, Bargmann CI (2001). Dynamic regulation of axon guidance.. Nat Neurosci.

[4] Benard C, Hobert O (2009). Looking beyond development: maintaining nervous system architecture.. Curr Top Dev Biol.

[5] Zallen JA, Kirch SA, Bargmann CI (1999). Genes required for axon pathfinding and extension in the C. elegans nerve ring.. Development.

[6] Sasakura H, Inada H, Kuhara A, Fusaoka E, Takemoto D (2005). Maintenance of neuronal positions in organized ganglia by SAX-7, a Caenorhabditis elegans homologue of L1.. Embo J.

[7] Pocock R, Benard CY, Shapiro L, Hobert O (2008). Functional dissection of the C. elegans cell adhesion molecule SAX-7, a homologue of human L1.. Mol Cell Neurosci.

[8] Benard CY, Boyanov A, Hall DH, Hobert O (2006). DIG-1, a novel giant protein, non-autonomously mediates maintenance of nervous system architecture.. Development.

[9] Bülow HE, Boulin T, Hobert O (2004). Differential functions of the C. elegans FGF receptor in axon outgrowth and maintenance of axon position.. Neuron.

[10] Aurelio O, Hall DH, Hobert O (2002). Immunoglobulin-domain proteins required for maintenance of ventral nerve cord organization.. Science.

[11] Benard C, Tjoe N, Boulin T, Recio J, Hobert O (2009). The small, secreted immunoglobulin protein ZIG-3 maintains axon position in Caenorhabditis elegans.. Genetics.

[12] Woo WM, Berry EC, Hudson ML, Swale RE, Goncharov A (2008). The C. elegans F-spondin family protein SPON-1 maintains cell adhesion in neural and non-neural tissues.. Development.

[13] Hammarlund M, Jorgensen EM, Bastiani MJ (2007). Axons break in animals lacking beta-spectrin.. J Cell Biol.

[14] Wang X, Kweon J, Larson S, Chen L (2005). A role for the C. elegans L1CAM homologue lad-1/sax-7 in maintaining tissue attachment.. Dev Biol.

[15] White JG, Southgate E, Thomson JN, Brenner S (1986). The structure of the nervous system of the nematode *Caenorhabditis elegans*.. Philosophical Transactions of the Royal Society of London B Biological Sciences.

[16] Hobert O, Bülow H (2003). Development and maintenance of neuronal architecture at the ventral midline of C. elegans.. Curr Opin Neurobiol.

[17] Hunt-Newbury R, Viveiros R, Johnsen R, Mah A, Anastas D (2007). High-throughput in vivo analysis of gene expression in Caenorhabditis elegans.. PLoS Biol.

[18] Freigang J, Proba K, Leder L, Diederichs K, Sonderegger P (2000). The crystal structure of the ligand binding module of axonin-1/TAG-1 suggests a zipper mechanism for neural cell adhesion.. Cell.

[19] Schurmann G, Haspel J, Grumet M, Erickson HP (2001). Cell adhesion molecule L1 in folded (horseshoe) and extended conformations.. Mol Biol Cell.

[20] Su XD, Gastinel LN, Vaughn DE, Faye I, Poon P (1998). Crystal structure of hemolin: a horseshoe shape with implications for homophilic adhesion.. Science.

[21] He Y, Jensen GJ, Bjorkman PJ (2009). Cryo-electron tomography of homophilic adhesion mediated by the neural cell adhesion molecule L1.. Structure.

[22] Meijers R, Puettmann-Holgado R, Skiniotis G, Liu JH, Walz T (2007). Structural basis of Dscam isoform specificity.. Nature.

[23] Sawaya MR, Wojtowicz WM, Andre I, Qian B, Wu W (2008). A double S shape provides the structural basis for the extraordinary binding specificity of Dscam isoforms.. Cell.

[24] Liu H, Focia PJ, He X (2011). Homophilic adhesion mechanism of neurofascin, a member of the L1 family of neural cell adhesion molecules.. J Biol Chem.

[25] Chen L, Zhou S (2010). “CRASH”ing with the worm: insights into L1CAM functions and mechanisms.. Dev Dyn.

[26] Soroka V, Kolkova K, Kastrup JS, Diederichs K, Breed J (2003). Structure and interactions of NCAM Ig1-2-3 suggest a novel zipper mechanism for homophilic adhesion.. Structure.

[27] Rougon G, Hobert O (2003). New Insights into the Diversity and Function of Neuronal Immunoglobulin Superfamily Molecules.. Annu Rev Neurosci.

